# Pharmacokinetics of plasma lopinavir/ritonavir following the administration of 400/100, 200/150, and 200/50 mg twice daily in HIV-negative volunteers

**DOI:** 10.1186/1758-2652-13-S4-P180

**Published:** 2010-11-08

**Authors:** AGA Jackson, A Hill, LJ Else, DJ Back, R Morley, R Puls, J Amin, E Lin, M Boffito

**Affiliations:** 1 Chelsea & Westminster Hospital, SSAT, London, UK; 2 University of Liverpool and Tibotec BVBA, Pharmacology Research Laboratories, Liverpool, UK; 3 University of Liverpool, Department of Pharmacology and Therapeutics, Liverpool, UK; 4 Chelsea & Westminster Hospital, London, United Kingdom; 5 University of New South Wales, Sydney, Australia

## Purpose of the study

Development and post-marketing data suggest that some licensed ARV doses could be reduced. We assessed the pharmacokinetics (PK) of lopinavir/ritonavir (LPV/r) following the administration of 3 different doses to HIV negative volunteers as a preliminary to the design of clinical trials to examine the safety and efficacy of novel dose regimens in HIV positive subjects.

## Methods

Following written consent, male and female volunteers were administered LPV/r 400/100 (2 LPV/r Meltrex 200/50 tablets; regimen 1), 200/150 (1 Meltrex tablet, 1 100mg ritonavir capsule; regimen 2), and 200/50 (1 Meltrex tablet; regimen 3) mg twice daily (BID) for 7 days sequentially. Each 7-day phase was separated by a 7-day wash-out period and LPV/r steady-state PK was assessed over 12 hours on the last day of each dosing phase (days 7, 21 and 35). PK parameters were compared using Phase 1 as reference by determining geometric mean ratios (GMR) and 90% confidence intervals (CI). Safety and tolerability were assessed throughout the study period.

## Summary of results

Twenty-two subjects (8 female) were enrolled and completed the study. GM PK parameters (90% CI) of the 3 doses are shown in Table 1.

**Table 1:** 

PK parameter	400/100 BID	200/150 BID	200/50 BID
	regimen 1	regimen 2	regimen 3

Lopinavir			

AUC0-12 (ng.h/mL)	99599 (87180-113787)	73603 (65121-83191)	45146 (39251-51927)

Cmax (ng/mL)	11965 (10400-13766)	8939 (8047-9930)	6404 (5648-7262)

C12h (ng/mL)	5776 (4884-6831)	4293 (3603-5115)	1749 (1419-2156)

			

Ritonavir			

AUC0-12 (ng.h/mL)	4664 (3808-5664)	10462 (8972-12200)	1625 (1390-1899)

			

LPV PK parameters in regimens 2 and 3 were lower: GMR (90%CI) AUC 0.74 (0.65-0.84) and 0.45 (0.40-0.51); Cmax 0.75 (0.66-0.85) and 0.54 (0.40-0.60); C12h 0.74 (0.62-0.89) and 0.30 (0.25-0.36). All subjects in regimens 1 and 2 had LPV concentrations above the suggested minimum effective concentration (MEC) of 1000ng/mL, 3 subjects receiving regimen 3 had lower concentrations. No serious adverse events were observed and as expected mild/moderate diarrhoea was the most common adverse effect.

## Conclusions

These PK data indicate that therapeutically relevant plasma concentrations of LPV can be achieved with lower administered doses and support further exploration of these lower LPV doses in properly designed randomised clinical trials. Preservation of therapeutically relevant LPV doses requires administration of higher doses of ritonavir. A new dose of LPV/r could lower cost and improve access in developing countries.

